# Bridging different realities - a qualitative study on patients’ experiences of preoperative care for benign hysterectomy and opportunistic salpingectomy in Sweden

**DOI:** 10.1186/s12905-020-01065-8

**Published:** 2020-09-11

**Authors:** Elin Collins, Maria Lindqvist, Ingrid Mogren, Annika Idahl

**Affiliations:** 1 Department of Clinical Sciences, Obstetrics and Gynecology, Umeå University, SE-901 87 Umeå, Sweden; 2 Department of Nursing, Umeå University, SE-901 87 Umeå, Sweden

**Keywords:** Health literacy, Hysterectomy, Opportunistic salpingectomy, Preoperative care, Preoperative consultation, Qualitative, Shared decision-making

## Abstract

**Background:**

Hysterectomy is a common procedure worldwide and removing healthy fallopian tubes at the time of hysterectomy (opportunistic salpingectomy) to possibly prevent ovarian cancer is increasing in frequency, but still controversial. The experiences and perceptions of women, eligible for the procedure, have not been previously investigated.

This study aims to, among women waiting to undergo hysterectomy, explore i) experiences and perceptions of self and healthcare in relation to their elective surgery, ii) perceptions of risks and benefits of hysterectomy, including opportunistic salpingectomy.

**Methods:**

A qualitative study, with focus group discussions including women < 55 years, planned for hysterectomy with ovarian preservation, was performed. Participants were recruited through purposive sampling from six gynecological departments in different parts of Sweden, including both country and university hospitals. Focus group discussions were conducted using a semi-structured interview guide, digitally recorded, transcribed verbatim and analysed by qualitative manifest and latent content analysis.

**Results:**

Twenty-one Swedish-speaking women participated. They were 40–53 years of age, reported varying educational levels, countries of birth and indications for hysterectomy. Analysis rendered a theme *“Bridging different realities”* over four categories*: “Being a woman today”, “Experiencing and managing body failure”, “Navigating the healthcare system”* and *“Processing continuously until surgery”*, including 17 subcategories. The participants displayed varying attitudes towards the significance of their uterus in being a woman. A vague understanding of their body was described, leading to fear related to the reasons for surgery as well as surgery itself. Participants described difficulties understanding and recalling information but also stated that insufficient information was provided. Perceptions of the risks and benefits of opportunistic salpingectomy varied. Involvement in decisions regarding the hysterectomy and potential opportunistic salpingectomy was perceived to be dependent on the counselling gynecologist.

**Conclusions:**

The theme Bridging different realities captures the complexity of women deciding on removal of their uterus, and possibly fallopian tubes. It also describes the women’s interactions with healthcare and perceived difference between the health professionals and the women’s perception of the situation, as viewed by the women. Bridging the different realities faced by patients is required to enable shared decision-making, through sufficient support from healthcare.

## Background

Hysterectomy is a common procedure with an annual estimate of over a million procedures performed worldwide [1]. Approximately 90% of these are performed for benign indications, with the aim of improving quality of life in women suffering predominantly from heavy bleeding or pain [1, 2]. In Sweden approximately 4200 hysterectomies were performed due to benign indications in 2019 [3].

A central concept in modern medicine is patient-centred care, stated as a goal by the World Health Organization (WHO) to achieve improved health outcomes [4]. In the WHO framework it is described that the individuals should be empowered by being involved in the co-production of healthcare. In the health-related empowerment concept a central part is health literacy, i.e. understanding your own body and health in order to make an informed decision [5]. An American study with women after having had a hysterectomy concludes a need for increased knowledge and empowerment in the women to be able to partake in the decisions regarding their surgery [6].

For the past 20 years data have accumulated supporting a theory that certain types of epithelial ovarian cancer originates in the fallopian tubes [7–9]. This has led to the introduction of the term “opportunistic salpingectomy”, which is the removal of presumed healthy fallopian tubes at the time of other benign intra-abdominal surgery with ovarian preservation, with the aim of potentially preventing ovarian cancer [10]. Opportunistic salpingectomy at the time of hysterectomy for benign indication is considered feasible which is noted as a widespread uptake of the procedure around the world [10–15]. In spite of this apparent acceptance in clinical practice there is still no consensus. Some national and international committees have issued statements with encouragement of discussing and recommending the procedure to women in the pre-surgical consultation prior to hysterectomy, while others stress the absence of evidence and call for studies in this field [16].

To our knowledge, no previous study has explored the experiences and perceptions of women related to consultation and preoperative care prior to hysterectomy, and in relation to making the decision of selecting opportunistic salpingectomy.

This study aims to, among women waiting to undergo hysterectomy, explore i) experiences and perceptions of self and healthcare in relation to their elective surgery, ii) perceptions of risks and benefits of hysterectomy, including opportunistic salpingectomy.

## Method

A qualitative study with focus group discussions (FGD) with women awaiting hysterectomy was conducted. Six departments of obstetrics and gynecology in Sweden were selected for recruitment of participants. The departments were selected on the basis of variation in geographical location in Sweden and health care levels, i.e. from county to university hospitals.

Approval from the heads of the included clinics was sought and granted before recruitment commenced. Adherence to data protection regulation in the EU, GDPR [17] was managed differently in each region, either the nurse coordinating the waiting list for surgery sought permission from the patient, or a legal representative, or a local research group, conducted a risk analysis of the research project. After approval at the local clinic, the contact information to eligible participants was given to the first author EC, who thereafter contacted eligible participants with an invitation to participate in the study. Oral and written informed consent were obtained before participation in the study. Inclusion criteria were women under the age of 55 years waiting to undergo hysterectomy for benign indications. Selection of participants was done by purposive sampling aiming at a variation in age, educational level and country of birth. Exclusion criteria were elective surgery on the ovaries, known hereditary predisposition for gynecological malignancy or insufficient knowledge in the Swedish language.

In typical management of care in Sweden, all pharmacological options should be exhausted before the patient is accepted for hysterectomy. The decision to perform hysterectomy is generally made at an appointment with a gynecologist, at which time the patient should be informed and provided the opportunity to ask her questions. The waiting time until hysterectomy is approximately 3 months in Sweden. A few days before the elective hysterectomy there is a final consultation with a gynecologist.

The FGDs were conducted while the participants waited for their elective hysterectomy. All focus groups were conducted at secluded locations outside of hospitals with refreshments being served. The aim was to include 6–8 participants in each group, but this was not achievable due to a limited number of women awaiting surgery at each clinic. In addition, late dropouts of participants due to personal matters resulted in 3–4 women participating in each FGD. Three eligible informants declined to participate once given oral information. The characteristics of the participants are described in Table 1.

**Table 1:** Background characteristics of participants

	Mean	Min - Max	n	%
Age (years)	47.9	40–53		
Pregnancies (n)	2.9	0–7		
Parity^a^	2	0–4		
Country of birth				
Europe			19	90.5
Asia			2	9.5
Education level				
University			11	52
High school			8	38
Compulsory school			2	10
Marital status				
Married/ Cohabiting			17	81
Living apart			2	9.5
Single			2	9.5
Indication for surgery^b^				
Menorrhagia/Metrorrhagia			9	43
Fibroids			15	71
Pelvic pain			3	14
Pelvic pressure			6	29
Endometriosis			1	4.8
Fear of cancer			2	9.5

^a^Number of children

^b^Indications according to the participants. The total percentage adds to > 100% due to combined indications

A semi-structured interview guide was constructed by the research team as a data collection tool (Additional data 1). Present at the FGDs were a moderator, a note-taker and at the first FGD one observer, all participants of the research team. Field notes of non-verbal communication were taken and discussed in the context of what was revealed in each group. Saturation of data was considered to have been reached after FGD 5, and FGD 6 was undertaken thereafter without any significant new information forthcoming. The FGDs were recorded digitally, lasting between 60 to 112 min and transcribed verbatim by first author EC, exempt transcripts of two FGDs which were written by a professional secretary and proofread by EC, while listening to the transcripts. Codes for FGD 1 to 3 were created by all authors individually and then discussed in meetings to reach consensus. For the remaining FGDs coding was done by EC and AI, with continued revision with the co-authors. The codes were then sorted into subcategories, and subcategories into categories in relation to their manifest content. Manifest and latent content analysis inspired by Graneheim and Lundman was applied [18]. The latent content of the materials was represented by an overarching theme.

## Results

The theme *Bridging different realities,* describes the complexity of a woman in deciding on hysterectomy, and in some cases salpingectomy. It also describes the women’s interactions with the healthcare system and the difference between the health professionals and the women’s perceptions of the situation, as perceived by the participants. The theme, categories and subcategories are presented in Table 2. Each subcategory is presented by a summary of the findings, and by supporting quotes from the participants in italics.

**Table 2:** Theme, categories and their subcategories

Theme	Categories	Subcategories
Bridging different realities	Being a woman today	Defining womanhood in removal of the uterus
		Being accomplished - an obstacle for own health
		Differing aspects on the significance of menopause
		Advocating for equality and just healthcare
	Experiencing and managing body failure	Suffering from a failing body
		Body image as preconception of function and dysfunction
		Cancer as a distant reality or a present fear
		Perspectives of health when experiencing a medical condition
	Navigating the healthcare system	Supportive and available healthcare
		Negligent and impersonal healthcare
		Balancing gain for yourself or future patients when participating in a study
		Wishing for optimal outcome but fully dependent
	Processing continuously until surgery	Information received and information perceived
		Accepting uncertainties or requiring more information
		Finding support or supporting in social networks
		The burden of waiting
		Accepting risks and trusting relief

### Being a woman today

This category has four subcategories *“Defining womanhood in removal of the uterus”, “Being accomplished - an obstacle for own health”, “Differing aspects on the significance of menopause”* and *“Advocating for equality and just healthcare”* together compiling a description of a contemporary woman’s view of herself and her perspectives on women’s health in awaiting surgery.

#### Defining womanhood in removal of the uterus

The participants described varying perspectives in the significance of the uterus. For some it was considered a vital part of being a woman, describing an intense longing to keep the body intact, while for others it was the uterus’ function which was essential. For the latter, when childbearing was completed hysterectomy could be performed without grief, although it raised thoughts about the finality of the reproductive phase.

*“My biggest issue facing it wasn’t the surgery itself, it’s what defines me as a woman [the uterus].”* (FGD 4)

*“For me it is not a big issue because it [hysterectomy] is something I have been striving towards. Since I’m done having children and it [the uterus] is just causing me problems and I don’t need it.”* (FGD 5)

#### Being accomplished - an obstacle for own health

The participants shared a desire to not cause inconvenience for others because of their own health issues, both related to the symptoms which were the indication for surgery and the expected convalescence period. It was described women postponing both the consultation and the surgery to manage work and social obligations. They also described a sense of guilt towards their partner and colleagues, and in relation to these matters also attempts to keep up the facade.

*“When you work you have to be positive, even if you’re not. It’s added pressure, I can sit on the toilet and cry like a baby. Because I can’t cry in front of my colleagues.”* (FGD 5)

The need to be accomplished also presented itself in how the participants experienced contacts with healthcare, with a desire to be prepared before gynecological examination, feeling inadequate for not understanding the reason for the symptoms and blaming themselves for not receiving sufficient information.

*“They [the doctors] haven’t talked about the other issue [the fallopian tubes]. But sometimes I think, should I have asked that question, should I have thought about it?*” (FGD 6)

#### Differing aspects on the significance of menopause

A multifaceted image of the significance of menopause was portrayed by the participants, ranging from the convenience of not menstruating, a view of menopause as natural and inevitable, and a step towards becoming a more knowledgeable woman.

*“I think I’m going to be a wise woman. I hope I’m on my way to becoming one … I’m very grateful to have reached the age I’m at and that I don’t have to be young today and fight against all the ideals.”* (FGD 4)

Opposing these positive perspectives were the attempts to avoid thinking about menopause, due to a perception of menopause as a step towards growing old and a negative image of women after menopause.

*“I think I’m going to feel old all of a sudden … I’m thinking of my mother, she went through it [menopause] when she was 50 and she became a different person. The woman she was, was no more. All that’s left is an old hag.*” (FGD 2)

#### Advocating for equality and just healthcare

Some participants conveyed frustration over a perceived lack of focus on female issues in society as well as in medicine, stating that research related to women’s health is insufficient and that this is due to a lower priority.

*“They [scientists] write what we know today. Right now, we don’t know if the fallopian tubes have any other function. Maybe we would have known more if we had done more research on it.” (FGD 4)*

There was a determination by participants to demand proper care and not accept unnecessary suffering. The frustration was not only directed at healthcare and the scientific community, but also at women in general, proclaiming a lack of knowledge and commitment from women in the society in regard to women’s health.

*“It’s [the lack of knowledge] a little shameful. Why don’t women know? Why don’t we talk about it?”* (FGD 1)

### Experiencing and managing body failure

This category includes four subcategories *“Struggling with a failing body”, “Body image as a preconception of function and dysfunction”, “Cancer as a present fear or a distant reality”* and *“Perspectives of health when experiencing a medical condition”* describing the intense suffering many women experienced before being accepted for surgery, and how their preconceptions and fears influenced their perspectives.

#### Struggling with a failing body

Many participants described profound effects on everyday life from their symptoms, often for an extended period of time, where measures from the healthcare services were insufficient. On some occasions own health had been put aside due to economics where being on sick-leave was considered too expensive to be an option. Pain, heavy bleeding and feelings of hopelessness were frequently described, all combined into a perception of a struggle.

*“The period for me is a nightmare, war, giving birth. I feel really bad when I have my period, I barely go outside. I don’t dare to go outside.”* (FGD 5)

*“It doesn’t work. I don’t even think I’m going to be alive for another 15 years if it is going to be like this.”* (FGD 6)

#### Body image as a preconception of function and dysfunction

Insufficient knowledge of anatomy and function of female genitalia as well as understanding of their diagnosis emerged in all groups. A consequence of this was an uncertainty of what to expect after surgery. What will fill the space after the uterus is removed?; Where does the penis go during intercourse?; What is the difference between ovary and fallopian tube?, were questions that were posed. The surgery was seen as something necessary but out of control.

*“When we talked about what to remove, I said ‘take out everything that is broken’, because it’s just a mess in there*.” (FGD 6)

#### Cancer as a present fear or a distant reality

In some participants the use of common gynecological diagnoses sparked fear of cancer, on some occasions relieved by more information from the doctor, in others the fear persisted.

*“That word sounds dangerous … Cyst, then it’s like cancer.”* (FGD 1)

Gynecological malignancies were viewed as a diffuse entity, for some the Pap smear was perceived as a screening method for all. Existing fear influenced the view on the extent of surgery. Participants with a fear of cancer wished to remove what was possible while the participants where cancer was perceived as distant wished for individually adapted surgery.

*“You can have cancer anywhere so why [worry about] specifically ovarian cancer?”* (FGD 4)

#### Perspectives of health when experiencing a medical condition

Health as a concept was described as being content with life and to be present and supportive towards family, irrespectively if there was an ongoing medical condition. Some participants emphasized the need to talk about health issues with close relatives and stressed the need of a functioning sexual relationship with their partner to stay healthy.

*“Contentment really can summarise it all. If I’m happy with what I get and have gotten in life and have an idea that it is going to continue … So not in terms of health and sickness or rich and poor, but being content.”* (FGD 4)

### Navigating the healthcare system

The four subcategories *“Negligent and impersonal healthcare”, “Supportive and available care”, “Balancing gain for yourself or future patients when participating in a study*” and *“Wishing for optimal outcome but fully dependent”* describe the difficulty in navigating the healthcare system where a dependency on the health professionals is central.

#### Negligent and impersonal healthcare

Several participants described a struggle to be taken seriously, and that repeated contacts with the clinic was required to get a date set for surgery. A feeling of being powerless in relation to the healthcare system was described; on some occasions with the perception of the clinics being in control of the situation but not conveying the information to the patients, in others distrust and a perceived need to monitor or intervene in the process, to be certain to acquire proper treatment. The healthcare provided was perceived to be in part dependent on the ability of the patient or relatives to navigate their way through the system.

*“I’ve come to think of [how difficult it must be for] old people who need to contact healthcare when not doing too well, when I think it’s hard, as someone who’s in a pretty good shape.”* (FGD 4)

*“I had more personal contact and information [previously]* … *Today everything is so computerised. It seems like the personal contact is disappearing, and your faith in the system disappears along with it.” (FGD 6)*

*“The [physician’s] pager beeped constantly and the phone rang and it was somebody [patient] who wasn’t doing so well, and it was like there were more urgent matters than a woman who might potentially have surgery.”* (FGD 5)

#### Supportive and available care

As a contrast to the previous subcategory, satisfaction with personal contacts and information provided by healthcare professionals were also reported. Receiving information by mail and filling out questionnaires online was stated to give a sense of trustworthiness and participants suggested that more information could be given electronically. The participants who were aware that routines could differ between clinics accepted it and decided to trust local recommendations. A shared decision-making with the gynecologist alleviated the decision and the wait for surgery.

*“You have to trust who you’re talking to, I’m sure there is a difference in opinions*.” (FGD 1)

*“[We should be grateful] that we live in a country where you can get help with these things, and you have people who communicate with you.”* (FGD 4)

#### Balancing gain for yourself or future patients when participating in a study

The question to partake in a clinical trial was met with ambivalence by some participants. The necessity of research was understood but participating in a study meant accepting an uncertainty of what could be the best approach. When informed of an ongoing clinical trial it was perceived difficult to understand; words like randomisation were not known by the general public, and lottery giving a negative association.

*“It’s good if you can participate in a study to help others, but at the end of the day you are the one you want it to work out for.”* (FGD 3)

*“I’ve given consent [to participate in a clinical trial] ... What happens, happens. It’ll contribute to science anyway*.” (FGD 2)

#### Wishing for optimal outcome but fully dependent

The participants reported a wish to receive individual recommendations regarding surgery, in some occasions to make an informed decision by themselves, and in others to directly follow the recommendations of the gynecologist. Some participants described being asked about removing their fallopian tubes although the gynecologists were not able to answer questions about the potential pros and cons. Others had not received the question on removal of the fallopian tubes and were unknowledgeable about what was planned, apart from hysterectomy. On many occasions the participants did not know the difference between fallopian tube and ovary, limiting the possibility of making a decision. The attitude towards opportunistic salpingectomy differed greatly, from removing only what is necessary to alleviate the symptoms, removing all parts possible or surrendering the decision to the gynecologist. A shared experience was that the consultation had been limited by short time and/or lack of information which impeded the ability to make an informed choice.

*“I don’t have the knowledge and cannot advise myself about pros and cons, if you say so, [give recommendations] then I have to rely on the professionals.”* (FGD 5)

*“I want to book another appointment and talk some more [about the surgery]. It wasn’t enough time [the previous visit] I don’t know … only 15-20 minutes.”* (FGD 3)

### Processing continuously until surgery

This category includes 5 subcategories; *“Information received and information perceived”, “Accepting uncertainties or requiring more information”, “Finding support or supporting in social networks”, “The burden of waiting”* and *“Accepting risks and trusting relief”,* and describes the continuous processing of information and decisions until the time of surgery.

#### Information received and information perceived

In many cases it was unclear for the women when the surgery would take place, what the surgical approach would be, and what could be expected during recovery. The participants could not state if they had been presented with the information or if they had not perceived what had been said. There was an awareness of the individual limits to what can be perceived, with some participants bringing the partner to the consultation as an aid to remember. To improve the processing of information there was a desire for information as pictorial material in addition to written information to bring home.

Several of the participants described a lack of information regarding surgical risks and the expected symptoms in recovery. Others were content with the information received, concerns regarding sexual function after surgery was addressed and they were confident that they would receive additional information if needed. There was an acceptance that the information given must be balanced not to cause unnecessary concern.

*“She [the physician] said the uterus. She probably described it but I can’t remember. She said what they were removing and how to stitch it up … it was hard to envision. I can’t really remember what she said, that’s what I got from that consultation.”* (FGD 5)

*“It comes naturally for a doctor and the anaesthesiologist … they do this all day. But it is not every day that I do this... Now I was a bit lost and a bit sad and all that, a bit in shock.”* (FGD 6)

#### Accepting uncertainties or requiring more information

For many participant’s multiple questions arose after their consultation, and their perceived trust in the health professionals affected how the participants handled their insufficient knowledge. Some were confident of receiving more information at a later stage and did not actively seek further information. Others avoided searching, out of fear of becoming more worried, but many participants described searching for more information online. Searching for more information gave varying results, some became relieved, while others became increasingly afraid where even different wording at different healthcare-websites was a cause for concern. This unaided gathering of information led to repeated phone-contacts and consultations at the clinics responsible for the participants.

*“I guess I’ll get more information later on.”* (FGD 6)

*“You read things [information online] you don’t know enough about and then you interpret it in a certain way and get worried and scared.”* (FGD 3)

#### Finding support or supporting in social networks

The participants described their social network as essential in managing the wait for surgery, often asking other women, who had experienced gynecological surgery, of their experiences. Some chose to bring their partners to the consultation as an aid to understand and remember the provided information but some to enable the partner to ask their questions and alleviate their worries. Many of the participants decided to partake in this study to meet other women in the same situation for mutual exchange.

*“My husband has been there [at the consultation], because when I get stressed out, I don’t know what I’m doing. He drew a picture for me the other day about what they said and what they meant about the fallopian tubes, so he had to explain it to me. You are not in a condition [to understand] even if you want to.” FGD1*

*“I wanted him to be there the whole time and hear the same information as me, and get to ask his questions.” FGD4*

#### The burden of waiting

An intense fear of what was to come was described, fearing both surgery and its potential consequences. Abstaining surgery was not seen as an option due to profound suffering from the symptoms and fear of what might be causing them.

*“Then I’m going to die but it doesn’t matter, anything is better … I’ll do whatever it takes anyhow, it doesn’t matter if it is frightening or not.” (FGD 2)*

Fear was reported due to unknown surgical approach, unknown time to surgery and uncertainty in how they would be contacted when it was time for surgery. Fear of anaesthesia and fear of a difficult convalescence where the information given regarding time to recovery was expected to be understated.

#### Accepting risks and trusting relief

The time waiting for surgery was in some participants mostly anticipation of improved quality of life, a trust in a functioning sexual relationship with the partner after surgery, and the end of suffering. There was a proclaimed trust in the healthcare to make the right decisions but also an acceptance that there could be negative side-effects to the surgery, and these risks were acceptable. It was expressed a trust in hysterectomy being a routine procedure, and a confidence in their own body to handle recovery, but also with an acceptance that healing might take some time.

*“I put my life in their hands and they see what’s needed and what isn’t.” (FGD 2)*

*“There is a risk of prolapse and everything that goes along with that, but I can’t have it the way it is either.”* (FGD 5)

*“I’m so happy, I’m so happy. I feel like the luckiest woman; I’m getting rid of it [the uterus].”* (FGD 5)

## Discussion

The theme *Bridging different realities* describes the complexity of women and their situation in relation to healthcare, when awaiting hysterectomy for benign indications. The main findings were a limited ability to make well-informed, independent decisions due to insufficient health literacy and a dependency on health professionals with insufficient information provided, as perceived by the participants. This is supported by previous studies including women after having had a hysterectomy, urging for more education prior to surgery [6]. If convalescence was discussed in the consultation, the problems were expected to be understated. These perceptions are supported by a study comparing information provided by gynecologists regarding expected recovery after gynecological surgery with actual return to various activities of daily living [19]. Experiences of failed medical treatment prior to being accepted for hysterectomy, which is common due to the standard of exhausting the pharmacological options before surgery, would also influence the trust of the information given. Insufficient knowledge while waiting for elective hysterectomy could lead to fear both regarding the indication for surgery as well as the surgery itself, although alleviated if there was a trust in the treating gynecologist. Patients’ expectations prior to elective surgery are known to correlate to quality of life after surgery [20]. Insufficient information combined with fear, which was expressed in this study, is likely to have a great impact on the experience of the surgery and recovery.

Health literacy, which was found insufficient for the participants in this study, is described in a model as the ability to access, utilise, understand and apply information in the domains of healthcare, disease prevention and health promotion [21]. All three domains are in part applicable in surgery for benign indications and to a greater extent in preventive surgery, i.e. opportunistic salpingectomy. The participants revealed a difficulty in understanding and remembering the information given, especially when feeling stressed, but also a lack of information received, and as described in previous studies participants turned to the internet [22], and to social contacts for further information [23]. Irrespective of educational level the participants generally portrayed a low basic knowledge in female anatomy and physiology, diminishing the ability to understand the consequences of hysterectomy.

The participants presented varying attitudes towards the significance of the uterus in their perception of their gender. For some individuals, where the uterus was seen as an organ with a specific function, it was perceived as uncomplicated to remove it when no longer functioning as expected, although the procedure demarcated the end of their reproductive period. For the participants where the uterus was seen as central to being a complete woman, the time-period leading up to surgery was a time of contemplating who they were going to be after the procedure, adding extra stress. Similarly, in a Norwegian study, persevering thoughts on not being a complete woman after hysterectomy was described if the sense of self was connected to the uterus [24]. Women experiencing a sense of being incomplete after hysterectomy have been described in studies from different parts of the world [6, 25]. For our participants, the fear of becoming incomplete as a woman after hysterectomy does not appear to stem from a fear of losing sexual ability. Irrespective of the importance they assigned the uterus, there was an anticipation of improvement in sexual function after surgery.

In our study the participants also shared a perception that involvement in decisions regarding their surgery was limited by a dependency on the counselling gynecologist, in regard to surgical method for hysterectomy as well as in regards to considering opportunistic salpingectomy. In Canada, a country with implementation of opportunistic salpingectomy into routine care [11], a qualitative study with gynecologists concluded a wish for more scientific evidence to be able to provide adequate information of opportunistic salpingectomy to their patients [26]. This is supported by participants in our study, describing experiences of the gynecologist not being able to explain the pros and cons of the procedure. In an American study including women after having had a hysterectomy, participants reported feeling they had no option but to follow the consultant’s recommendation, even if given an alternative [6].

Our results of the participants’ perceived inability to make decisions regarding own health due to insufficient health literacy, and inadequate support from healthcare brings into focus what is required to achieve shared decision-making between patient and health professional. A comprehensive article, describing shared decision-making in situations with unclear evidence, states that for the patients to construct a preference in a complex situation it requires more than being presented with information and being invited to choose [23]. Self-awareness of the medical professional is essential due to the risk of involuntary coercion towards preferences of the counsellor if not consideration is payed to individual aspects for the patient [23]. This is important in any medical consultation and even more so in preventive medicine. Opportunistic salpingectomy, as a preventive measure with unclear preventive size-effect and unclear long-term consequences according to a recent Cochrane report [27], requires that women undergoing the surgery be fully informed and enabled to partake in the decisions.

### Strengths and limitations

To ensure trustworthiness the central concepts of dependability, confirmability, transferability and credibility were addressed at all stages of the study [18]. Credibility was addressed by triangulation within the research group, with varying backgrounds in women’s health, in the preparation of the study and on all levels of analysis, until consensus was reached. After performed FGDs, participants in all groups revealed appreciation in sharing experiences with others, indicating a secure setting inviting the participants to share their reality and supporting the dependability of the results. Dependability was also strengthened by parallel construction of codes of different members of the research group. Confirmability was addressed by the presentation of quotes to support the abstraction of each subcategory, and field-notes were taken throughout the process of data-collection and analysis and then utilised in the analysis.

The composition of the research team with a consultant (first author) and senior consultants in obstetrics and gynecology, and a midwife, all female health professionals working within different aspects of women’s health, created a preunderstanding of the issues facing these participants but could also be seen as a possible limitation due to preconceptions.

The FGDs were conducted at a time while most of the women had not yet received the date of surgery, nor met for the final consultation. The final consultation is usually conducted only days before being admitted to hospital and the participants were likely to receive more information at this point. It can be argued that the woman (patient) should be informed early in the process to be able to weigh the different options during the waiting period, to enable shared decision-making, and to reduce unnecessary concerns.

The study was performed in Sweden, a highly secular country [28], with the availability of advanced healthcare. The participants’ views on certain aspects, such as the interaction with healthcare, the significance of the uterus and their views on menopause is likely to differ in other cultural settings [29]. The central findings in this study are a lacking health literacy, a dependency on health professionals and a limited ability to make independent decisions regarding health interventions, all of which we assess to be global health issues.

## Conclusions

The theme *Bridging different realities,* portrays the complexity of the situation for a woman in deciding on the removal of her uterus, and possibly her fallopian tubes. It also describes the participant’s perceived difference in how healthcare professionals and patients view the problems facing these women. Being aware of the complexity of these patients, and the coherent requirements of sufficient support from the healthcare system, is central in regard to any consultation prior to surgery. In the decision of performing a preventive surgery, e.g. opportunistic salpingectomy, it is even more important that the individual is able to make an informed choice. To support the decision process, the patient needs to receive information in an adequate time and manner. Of great importance is also acknowledging and addressing an insufficient health literacy, as observed in this study. A change in preoperative care and consultation is needed if the aim is to achieve shared decision-making, a step towards patient-centred care to improve health outcomes as recommended by the WHO.

## Supplementary information

**Additional file 1.** : Interviewguide.pdf, semi-structured interview guide used as a data collection tool.

## Data Availability

Not applicable.

## Abbreviations

WHO: World Health Organization
FGD: Focus group discussion
GDPR: General Data Protection Regulation
EU: European Union
