# Supplementary material for: Bridging different realities - a qualitative study on patients’ experiences of preoperative care for benign hysterectomy and opportunistic salpingectomy in Sweden
Source: BMC Womens Health. 2020 Sep 11;20:198. doi: 10.1186/s12905-020-01065-8 (PMC7488533; doi:10.1186/s12905-020-01065-8)
Supplement: Supplementary file 1 — Additional file 1. : Interviewguide.pdf, semi-structured interview guide used as a data collection tool. [file 12905_2020_1065_MOESM1_ESM.pdf]

INTERVIEWGUIDE

1. If you feel comfortable, could you please start by introducing yourself by telling us your first name and the reason for your hysterectomy?

2. Could you tell us about the consultation when the decision to perform surgery was made?
    a. What information were you given about the planned surgery?
    b. What were you told about the pros and possible cons about also removing the fallopian tubes?
    c. From which other sources have you sought information about the surgery?
    d. What sources do you consider trustworthy?

3. What is health?
    a. A person who feels well and has good health- what defines such a person to you?
    b. What are your associations to femininity? What defines a person as a woman?

4. What are your thoughts about the planned surgery?
    a. What are your thoughts regarding the pros of removing the uterus?
    b. Risks about surgery, what are your thoughts?
    c. Some women feel that their sense of femininity is affected by removing parts of the female genital organs? What are your reflections on that?
    d. We know that removing the uterus can lead to a earlier menopause and there are some indications that removing the fallopian tubes can affect the ovarian function further. What are your reflections on that?
    e. How do you think your sex-life could possibly be affected by the surgery?

5. When you approach menopause you can have symptoms like changed bleeding pattern but also symptoms of changed levels of hormones, e.g. flushes, changes in skin and mucosa, changes in mood. How do you envision menopause for yourself?
    a. Does/ Will it change how you see yourself?
    b. How do you think life can be altered?
    c. Have you or any acquaintance experienced substantial changes in menopause? Problems? Relief?
    d. Do you expect sex-life to be affected by menopause?
    e. Do you expect menopause to affect health in general?

6. What are your perceptions on your own risk of being diagnosed with ovarian cancer?
    a. How do you think the disease could affect your life?
    b. If you have a relative/ acquaintance who has been diagnosed, how has it affected her life?
        i. How does that affect your thoughts about own risk for ovarian cancer?

7. Earlier, the standard at hysterectomy has been to leave the fallopian tubes. Today there is a discussion about removing them at the time of surgery. The reason for this is that there are indications that removing the fallopian tubes might prevent certain types of ovarian cancer, although not scientifically proven yet. What speaks against removing the fallopian tubes is an

apprehension about negatively affecting ovarian function thereby possibly leading to premature menopause and a slightly more extensive surgery. Have you any reflections regarding this?

   a. There is an ongoing study in Sweden which is investigating if the fallopian tubes can be removed without risk of consequences and if it prevents ovarian cancer. In this study one is randomised to keep or remove the fallopian tubes. What are your thoughts on randomisation/ lottery regarding surgery in a study?
   b. The preferred surgery (i.e. removing the fallopian tubes or not at hysterectomy) differs around the world and also within Sweden, with some places favouring removal and some not. Have you any reflections regarding this?
   c. If it were to be revealed that the surgery does not prevent cancer but does negatively impact ovarian function, what are your thoughts on this?

8. Miscellaneous
   a. Is there any more thoughts or feelings regarding the subject that we haven´t discussed today?
   b. How did you perceive today’s focus group discussion?
   c. Are you bringing anything with you from today’s discussions?
